# *Mycobacterium ulcerans* Disease: Experience with Primary Oral Medical Therapy in an Australian Cohort

**DOI:** 10.1371/journal.pntd.0002315

**Published:** 2013-07-18

**Authors:** N. Deborah Friedman, Eugene Athan, Andrew J. Hughes, Masoomeh Khajehnoori, Anthony McDonald, Peter Callan, Richard Rahdon, Daniel P. O'Brien

**Affiliations:** 1 Department of Infectious Diseases, Barwon Health, Geelong, Australia; 2 Deakin Medical School, Geelong, Australia; 3 Department of Plastic Surgery, Barwon Health, Geelong, Australia; 4 Department of Medicine and Infectious Diseases, Royal Melbourne Hospital, University of Melbourne, Melbourne, Australia; 5 Manson Unit, Médecins Sans Frontières, London, United Kingdom; University of California San Diego School of Medicine, United States of America

## Abstract

**Background:**

*Mycobacterium ulcerans* (MU) is responsible for disfiguring skin lesions and is endemic on the Bellarine peninsula of southeastern Australia. Antibiotics have been shown to be highly effective in sterilizing lesions and preventing disease recurrences when used alone or in combination with surgery. Our practice has evolved to using primarily oral medical therapy.

**Methods:**

From a prospective cohort of MU patients managed at Barwon Health, we describe those treated with primary medical therapy defined as treatment of a *M. ulcerans* lesion with antimicrobials either alone or in conjunction with limited surgical debridement.

**Results:**

From 1/10/2010 through 31/12/11, 43 patients were treated with exclusive medical therapy, of which 5 (12%) also underwent limited surgical debridement. The median patient age was 50.2 years, and 86% had WHO category 1 and 91% ulcerative lesions. Rifampicin was combined with ciprofloxacin in 30 (70%) and clarithromycin in 12 (28%) patients. The median duration of antibiotic therapy was 56 days, with 7 (16%) receiving less than 56 days. Medication side effects requiring cessation of one or more antibiotics occurred in 7 (16%) patients. Forty-two (98%) patients healed without recurrence within 12 months, and 1 patient (2%) experienced a relapse 4 months after completion of 8 weeks of antimicrobial therapy.

**Conclusion:**

Our experience demonstrates the efficacy and safety of primary oral medical management of MU infection with oral rifampicin-based regimens. Further research is required to determine the optimal and minimum durations of antibiotic therapy, and the most effective antibiotic dosages and formulations for young children.

## Author Summary

*Mycobacterium ulcerans* (MU) is responsible for disfiguring skin infections which are challenging to treat. The recommended treatment for MU has continued to evolve from surgery to remove all involved tissue, to the use of effective combination oral antibiotics with surgery as required. Our study describes the oral medical treatment utilised for consecutive cases of MU infection over a 15 month period at our institution, in Victoria, Australia. Managing patients primarily with oral antibiotics results in high cure rates and excellent cosmetic outcomes. The success with medical treatment reported in this study will aid those treating cases of MU infection, and will add to the growing body of knowledge about the relative roles of antibiotics and surgery for treating this infection.

## Introduction

Buruli ulcer, also known as Bairnsdale ulcer, Daintree ulcer, or Mossman ulcer is a necrotising infection of skin and subcutaneous tissue caused by *Mycobacterium ulcerans* (MU) [1]. The major burden of disease is found in tropical climates, but cases have been reported from 33 countries worldwide [2]. *M.ulcerans* infection has become endemic on Victoria's Bellarine Peninsula in South-eastern Australia [1]

Surgical management was traditionally the standard treatment for *M. ulcerans* disease [1], [3], however recurrence of infection ensued in 17–32% of patients [4], [5]. Evidence of the effectiveness of antimicrobials used alone or combined with surgery [4]–[7], has led to an evolution of our standard treatment practice over the last 15 years to now comprise combination antimicrobial therapy with limited surgical debridement when required [5], [8]. Improved awareness in our region about MU lesions in the community has resulted in earlier referral of patients with smaller lesions to our department at Barwon Health [9].

We previously advocated for further studies using oral antibiotic regimens [7]. Here we describe results from an observational cohort of patients from South-eastern Australia managed with primary oral medical treatment for *Mycobacterium ulcerans* infection.

## Methods

### Ethics Statement

This is an observational cohort study, approved by Barwon Health's Human Research and Ethics Committee. All previously gathered human medical data were analysed anonymously.

Data on all confirmed *M. ulcerans* cases managed at Barwon Health has been collected prospectively since January 1998. From October 2010, our standard treatment practice for initial *M. ulcerans* lesions has comprised combination antimicrobial therapy, with limited surgical debridement performed to aid wound healing. The data extracted from medical records included; patient demographics and co-morbid conditions, details of the MU lesion, regimen and duration of antimicrobial therapy, and details of surgical procedures. Cases treated between October 2010 and December 2011 with 12 months follow-up were included in this cohort.

### Definitions

A *M. ulcerans* case was defined as the presence of a lesion clinically suggestive of *M. ulcerans* plus any of (1) a culture of *M. ulcerans* from the lesion, (2) a positive Polymerase Chain Reaction (PCR) from a swab or biopsy of the lesion, or (3) histopathology of an excised lesion showing a necrotic granulomatous ulcer with the presence of acid-fast bacilli (AFB) consistent with acute *M. ulcerans* infection.

The anatomical location of a *M. ulcerans* lesion was described as distal if it was on the elbow or below, or on the knee or below [7], [10].

Primary medical treatment was defined as treatment of a *M. ulcerans* lesion with either antimicrobials alone or antimicrobials in conjunction with limited surgical debridement. Drug dosages for adults included ciprofloxacin 500 mg twice daily, moxifloxacin 400 mg daily, rifampicin 10 mg/kg/day (up to a maximum of 600 mg daily), and clarithromycin 500 mg twice daily.

Paradoxical reactions were defined by the presence of one or both of the following features: a) clinical: an initial improvement on antibiotic treatment in the clinical appearance of a *M. ulcerans* lesion followed by deterioration of the lesion or its surrounding tissues, or the appearance of a new lesion(s), and b) histopathology: examination of excised tissue from the clinical lesion showing evidence of an intense inflammatory reaction consistent with a paradoxical reaction [11].

Limited surgical debridement was defined as curettage of the lesion or a minor excision to remove excess granulation tissue and to debride ulcer margins, with or without the use of a split skin graft (SSG). Limited surgical debridement was undertaken primarily to remove necrotic tissue from the MU lesion in order to promote healing by secondary intention.

Patients who underwent extensive surgery (defined as complete excision of the entire lesion including margins of non-necrotic tissue, with either direct closure or the use of a SSG or a vascularised skin and tissue flap for reconstruction or to cover the defect) were excluded from the formal analysis.

Criteria for primary medical therapy in our practice includes; patient willingness to take antimicrobials, and no contraindications to antimicrobial therapy (for example; drug interactions, or severe liver disease).

Criteria for complete surgical excision include factors such as; a lesion suitable for removal with direct wound closure, need for reconstruction to close a skin defect via flap or SSG, patient unable or unwilling to take antimicrobials, and patient or surgeon preference.

Definitions of treatment success, treatment failure, disease recurrence, and immune suppression were as published previously [2], [5].

A complication of medical therapy was defined as an adverse event attributed to an antibiotic that required cessation of that medication.

Data was collected using Epi-Info 6 (CDC, Atlanta) and analysed using STATA 12 (StataCorp, Texas, USA). Categorical values were compared using the Mantel-Haenszel test and median values were compared using the Mann-Whitney test.

## Results

From 1/10/2010 through 31/12/11, there were 54 patients with MU infection managed at Barwon Health. From this cohort 11 patients (20%) were excluded from further analysis; 3 patients underwent primary complete surgical excision alone, and 8 patients were prescribed antimicrobials but also underwent complete surgical excision. There were no significant differences in baseline characteristics of those excluded from those included in the analysis (table 1).

**Table 1:** Baseline characteristics of patient cohort.

Characteristics	Medical Management (43 patients)	Surgical Management (11 patients)	p-value
Median Age (IQR)	50.2 years (26.7–75.1 years)	53.3 years (40.2–64.5 years)	0.76
Male gender	28 (65.1%)	5 (45.5%)	0.23
Median duration symptoms prior to diagnosis (IQR)	35 days (21–75 days)	60 days (30–90 days)	0.24
Lesion type			
Ulcer	39 (90.7%)	11 (100%)	0.57
Nodule	3 (7.0%)	0 (0%)	0.57
Oedematous	1 (2.3%)	0 (0%)	0.57
WHO stage			
1	37 (86.1%)	9 (81.2%)	0.56
2	4 (9.3%)	2 (18.8%)	0.56
3	2 (4.7%)	0	0.56
Lesion location			
Lower limb	29 (65%)	4 (36.4%)	0.06
Upper limb	14 (35%)	7 (63.6%)	0.06

Forty-three (80%) patients were therefore included in this analysis. Baseline characteristics can be seen in table 1. All patients were primarily managed as outpatients. The majority of patients were male (65%), and the median age was 50.2 years (range 1.5–87.9 years). Lesions were ulcerative in 91% and WHO stage 1 in 86% of patients. All patients resided in areas of the Bellarine peninsula where MU is endemic; the majority residing in Point Lonsdale (36%) and Barwon Heads (29%). Four patients (9%) had known co-morbidities including diabetes (2), immune suppression (1), and malignancy (1). No patients were known to be HIV-infected though active screening was not performed.

Antimicrobial regimens were all rifampicin-based. Rifampicin was combined with; ciprofloxacin in 30 (70%) patients, clarithromycin in 12 (28%) patients, and moxifloxacin in 1 (2%) patient. The median duration of therapy was 56 days (range 28 to 91 days). Seven of 43 patients (16%) received less than 56 days of therapy. Antibiotic-associated complications requiring cessation of one or more antibiotics occurred in 7 of 43 patients (16%). Two patients developed complications attributed to the combination of rifampicin and ciprofloxacin, 2 patients developed complications attributed to ciprofloxacin, 1 patient developed complications due to moxifloxacin, and 2 patients developed complications due to rifampicin. The most common complications were gastrointestinal upset in 4 patients, joint aches in 2 patients, hepatitis in 2 patients, tendonitis in 1 patient and thrombocytopenia in 1 patient. Of the 11 patients who underwent complete excision and were excluded from the study cohort, 2 of those 8 who took antibiotics developed complications.

Nine of 43 patients (21%) developed paradoxical reactions a median of 34 days after antibiotic therapy initiation (IQR 20–92 days).

Five of 43 (12%) medically managed patients also underwent limited surgical debridement, and 3 of these procedures involved a SSG for coverage of the defect.

**Figure 1:**
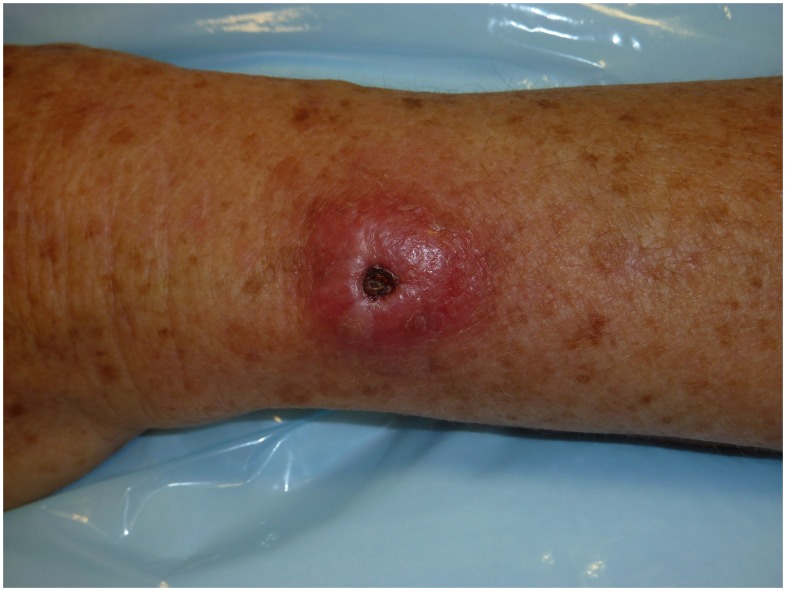
*Mycobacterium ulcerans* lesion at commencement of antimicrobial therapy.

**Figure 2:**
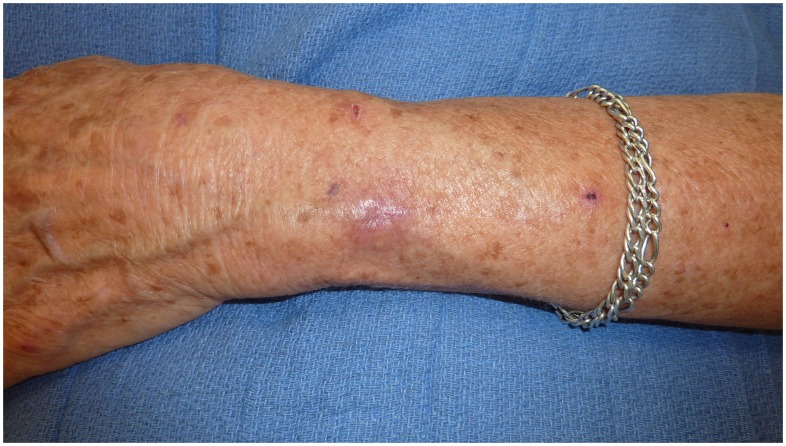
*Mycobacterium ulcerans* lesion 9 months after antimicrobial therapy commenced.

Overall, 42 of 43 patients (98%) were cured with primary medical therapy. Cosmetic outcomes were excellent in these medically managed MU cases (Figures 1 & 2). One patient failed primary medical therapy. This patient was a 17 month-old boy who presented with a nodular MU lesion on his arm of 1 cm diameter. He completed 56 days of rifampicin (10 mg/kg/day) with clarithromycin (13 mg/kg/day in twice daily dosing) both in liquid formulations with initial reduction in the size of the lesion. Four months after antimicrobials were completed the lesion enlarged and was debrided. Tissue from the debridement was culture positive for *M. ulcerans*. The patient was re-treated with rifampicin (10 mg/kg/day) and clarithromycin (13 mg/kg/day in twice daily dosing) both in liquid formulations, with subsequent ulceration of the nodule. A paradoxical reaction was diagnosed 4 weeks after re-treatment commenced based on the clinical deterioration of the lesion and was treated with prednisolone at a dose of 1 mg/kg for 4 weeks. The ulceration progressed and ultimately extended over an area of 12×5 cm and healed fully by secondary intention over a period of 9 months (16 months after initial treatment).

## Discussion

Combination antimicrobial therapy is now routinely recommended to treat *Mycobacterium ulcerans* infection with or without the addition of surgical intervention [4]–[6], [12]. Here we describe our experience with primary medical management of MU infection in South-eastern Australia using oral rifampicin-based regimens and we demonstrate the efficacy and safety of this approach with healing of lesions within 12 months of therapy initiation in 98% of cases, an acceptable toxicity profile and good cosmetic results.

In our study, the median duration of therapy was 8 weeks, although over 15% of patients completed less than 56 days of therapy. Previous data from Etuaful and others demonstrated viable MU organisms in tissue after 2 weeks of therapy, but not after 4, 8 or 12 weeks of therapy [13]. We reported that mycobacterial cultures were positive in excised specimens from the majority of patients (55%) who received less than 14 days of antibiotic therapy, but in only 1 of 8 patients (12.5%) who received more than 14 days of treatment [5]. Although WHO recommends combined antibiotic treatment for 8 weeks as first-line therapy for all *M. ulcerans* lesions [12], shorter durations of antimicrobials may be adequate in selected patients and further research should be performed to explore this possibility [7].

In the recent MU antimicrobial therapy literature, some differences exist in the inclusion or exclusion of patients who underwent surgery. In addition, there are likely geographic differences in surgical practice. In Chauty's medically treated cohort, 37% of patients underwent limited surgery, (and 13% underwent extensive surgery), while in Sarfo's study, 5% of patients underwent SSG while the remaining 95% healed without surgery [4], [7]. In the present study, 12% of medically managed patients underwent limited surgery (including minimal debridement and SSG) to promote wound healing and closure, and we excluded patients who underwent extensive surgery.

The selection of patients for medical therapy has not been clearly defined, and size of the MU lesion may or may not be a useful criterion. While smaller (WHO category 1) lesions are likely to achieve cure with medical therapy, they are also likely to be suitable for surgical excision with direct closure. WHO category 1 lesions have been shown to heal with medical therapy after a median of 12–18 weeks [4], [6]. In contrast, large lesions (WHO categories 2 and 3) may require more disfiguring surgery to achieve healing, while medical therapy for these lesions may result in slow but gradual shrinkage and secondary healing. The healing of larger lesions (WHO categories 2 and 3) is variable, and occurs anywhere from 11–15.5 weeks [4], to 30 weeks after initiation of antimicrobials [6]. In our cohort, the majority of patients (81%) who underwent surgical excision had WHO category 1 lesions. In some other studies of medical therapy for MU, researchers have only included lesions less than 10 cm in cross-sectional diameter [6], [7], while we and others have included lesions of all sizes [4].

Antimicrobial therapy for MU appears to be safe and reasonably well tolerated. In a study of rifampicin and streptomycin therapy, treatment was well tolerated with only 3 of 160 patients (1.8%) developing side effects [4]. In a pilot study of oral chemotherapy for MU infection, the authors described rifampicin and clarithromycin in combination as well-tolerated with no adverse effects [7]. It is worthy to note that in their cohort of 30 patients, all were hospitalized during therapy, which would be expected to both boost adherence and manage minor side effects more easily [7]. In our cohort, 7 of 43 patients (16%) ceased one or more antibiotics due to side effects. This rate of side effects is in keeping with rates our group previously described for rifampicin, clarithromycin and ciprofloxacin, which were higher than rates reported in younger populations in Africa [4]. We believe the older median age of our cohort may explain reduced drug tolerability, but that overall the side effect profile is acceptable [5].

Antimicrobial therapy has been demonstrated to improve MU lesion healing and prevent relapse. In non-controlled trials, rifampicin-based regimens in conjunction with clarithromycin [7], or a fluoroquinolone [5], [11], have demonstrated success in managing MU infection. Our antimicrobial treatment success rate of 98% is in keeping with other studies of medical management of MU [4], [6], [7]. In Sarfo's study of 160 patients, and Chauty's cohort of 30 medically managed patients there were no recurrences or relapses during 1 year of follow-up after treatment initiation [6], [7]. In Nienhuis's study, there was a 1.4% recurrence rate [4], and in our cohort of 43 patients we describe one relapse in a patient with a nodular form of MU infection.

Although the cure rates described in our Australian cohort are comparable to studies conducted in Africa [4], [6], [7], it is possible these outcomes could be influenced by identified small differences in *M. ulcerans* genomic sequences. *M. ulcerans* strains worldwide produce a very restricted repertoire of mycolactones, although Australian strains characteristically produce predominantly mycolactone C compared with predominantly mycolactone AB produced in Africa [14]. In addition, *M. ulcerans* has evolved by acquiring a plasmid (pMUM) and other independent genomic changes within strains from different areas to produce region- specific phenotypes and genotypes [15], [16]. However, recent analysis has revealed that Buruli ulcer in Africa and Australia is caused by one distinct lineage of mycolactone-producing *Mycobacteria* comprising a highly clonal group [17]. This close genetic relationship suggests that strain differences are minimal and there is currently no evidence to suggest that these strain differences significantly influence responses to therapy or cure rates. There are however some differences between patient populations in our study and those conducted in Africa. For example, the median age in our cohort was 50 years compared with 12 years in studies conducted in Ghana [4], [6]. In our study over 90% of lesions were ulcerated, compared with 36–70% in African cohorts [4], [6], [7], and none of our patients were HIV infected compared with 2% in Ghana [6]. Finally, there are possible unmeasured differences in nutrition and innate response to infection that may affect cure rates in different geographic regions.

There are a number of potential reasons for the relapsed case seen in our study. Firstly, it may relate to the nodular form of disease as medical management is thought to be sufficient for ulcerative lesions but some believe that most non-ulcerative lesions require additional surgery [7]. It may be more difficult to sterilize lesions that don't ulcerate and discharge the underlying necrotic material containing large numbers of mycobacteria. Secondly, it is possible that the ulceration of this nodule was part of the natural history of nodular MU disease, as has been described by Nienhuis and others [18], whereby most nodules ulcerate during the healing process. However the fact that the area of ulceration was significantly larger than the initial nodule and the lesion remained culture positive 6 months after antibiotics commenced argue against this. Finally, it is possible that the liquid preparations of antimicrobials used in this case contributed to treatment failure. It has been shown that the bioavailability of rifampicin varies depending on the drug preparation [19]. Furthermore, experience suggests that the use of syrups in young children can adversely affect adherence potentially leading to under dosing. Thirdly, it may be that recommended doses of antimicrobials in young children are inadequate, especially as no specific research has been performed on drug levels in this age-group for MU treatment. Research in children hospitalized for treatment of tuberculosis has found that their serum rifampicin concentrations were considerably less than the suggested lower limit for 2-hour rifampicin concentrations in adults after receiving standard rifampicin dosages similar to those used in adults [20]. This study however utilized fixed-dose combination tablets formulated for paediatric use, which is not comparable to the preparation used in our patient.

There are limitations to our study; firstly it's observational design and the exclusion of 20% of patients during the study period as they underwent excisional surgery. Although there were no significant differences in baseline characteristics between this group and those included, it is possible that there were other unknown confounders that may have introduced a selection bias and affected the validity of our findings [4], [6], [7]. Secondly, we are unable to detail the exact timing of healing of MU lesions, which would be valuable information. Finally, most of the lesions treated in our study (86%) were WHO Category 1 lesions and therefore the strength of our evidence for treatment success for lesions of larger size is weaker, although there have been descriptions in the literature of good success rates with medical therapy for lesions larger than 5 cm [6], [7].

### Conclusions

Our experience demonstrates the efficacy and safety of primary oral medical management of *M. ulcerans* in an Australian cohort. Further research is required to determine the optimal and minimum durations of antimicrobial therapy and the most effective dosages and formulations of antimicrobials for young children.
